# Everolimus Alleviates Renal Allograft Interstitial Fibrosis by Inhibiting Epithelial-to-Mesenchymal Transition Not Only *via* Inducing Autophagy but Also *via* Stabilizing IκB-α

**DOI:** 10.3389/fimmu.2021.753412

**Published:** 2022-01-24

**Authors:** Zeping Gui, Chuanjian Suo, Jun Tao, Zijie Wang, Ming Zheng, Shuang Fei, Hao Chen, Li Sun, Zhijian Han, Xiaobing Ju, Hengcheng Zhang, Min Gu, Ruoyun Tan

**Affiliations:** ^1^ Department of Urology, the Second Affiliated Hospital With Nanjing Medical University, Nanjing, China; ^2^ Department of Urology, The First Affiliated Hospital With Nanjing Medical University, Nanjing, China; ^3^ Transplantation Research Center, Brigham and Women’s Hospital, Harvard Medical School, Boston, MA, United States

**Keywords:** chronic renal graft dysfunction, renal allograft interstitial fibrosis, everolimus, autophagy, EMT

## Abstract

Chronic allograft dysfunction (CAD) is the major cause of late graft loss in long-term renal transplantation. In our previous study, we found that epithelial–mesenchymal transition (EMT) is a significant event in the progression of renal allograft tubulointerstitial fibrosis, and impaired autophagic flux plays a critical role in renal allograft fibrosis. Everolimus (EVR) has been reported to be widely used to prevent the progression of organ fibrosis and graft rejection. However, the pharmacological mechanism of EVR in kidney transplantation remains to be determined. We used CAD rat model and the human kidney 2 (HK2) cell line treated with tumor necrosis factor-α (TNF-α) and EVR to examine the role of EVR on TNF-α-induced EMT and transplanted renal interstitial fibrosis. Here, we found that EVR could attenuate the progression of EMT and renal allograft interstitial fibrosis, and also activate autophagy *in vivo*. To explore the mechanism behind it, we detected the relationship among EVR, autophagy level, and TNF-α-induced EMT in HK2 cells. Our results showed that autophagy was upregulated upon mTOR pathway inhibition by EVR, which could significantly reduce expression of TNF-α-induced EMT. However, the inhibition of EVR on TNF-α-induced EMT was partly reversed following the addition of autophagy inhibitor chloroquine. In addition, we found that TNF-α activated EMT through protein kinase B (Akt) as well as nuclear factor kappa B (NF-κB) pathway according to the RNA sequencing, and EVR’s effect on the EMT was only associated with IκB-α stabilization instead of the Akt pathway. Together, our findings suggest that EVR may retard impaired autophagic flux and block NF-κB pathway activation, and thereby prevent progression of TNF-α-induced EMT and renal allograft interstitial fibrosis.

## Introduction

Renal transplantation has long been regarded as the best therapeutic intervention for patients with end-stage organ failure in recent years (1). While immunosuppression induced by the calcineurin inhibitors tacrolimus (FK506) or cyclosporine A (CsA) has significantly improved short-term graft survival in kidney transplantation, a satisfactory rate of long-term renal allograft survival has not been achieved over the last two decades (2). Chronic allograft dysfunction (CAD), formerly known as chronic allograft nephropathy, is the most prevalent cause of late renal graft loss in kidney transplantation and has a significant impact on the long-term survival of the transplanted kidney (3).

CAD is characterized by glomerulosclerosis, peritubular capillary loss, chronic inflammation and interstitial fibrosis/tubular atrophy (IF/TA) (4). Interstitial fibrosis is an essential factor in determining the outcome of the renal allograft, although the underlying mechanisms are unknown. Several studies, including our previous research, have demonstrated that renal graft interstitial fibrosis is characterized by the accumulation of extracellular matrix (ECM), as a result of increasing fibroblasts and myofibroblasts (5, 6). Myofibroblasts are formed from a variety of distinct cell types, including epithelial cells, endothelial cells, bone marrow-derived fibroblasts, and microvascular pericytes (7, 8). The epithelial–mesenchymal transition (EMT) is the process through which epithelial cells gradually lose their markers, such as E-cadherin, and gain mesenchymal markers such as α-smooth muscle actin (α-SMA) (9). It is divided into several subtypes, with type 2 EMT being involved in tissue regeneration, wound healing, and organ fibrosis (10). EMT occurs as an inherent mechanism to support neovascularization and cardiac healing; this could imply that EMT plays a prosthetic and protective role in pathogenesis following myocardial infarction (11). However, Andugulapati et al. demonstrated that they could inhibit pulmonary fibrosis *in vitro* and *in vivo* by suppressing the transforming growth factor-β (TGF-β)-mediated EMT, myofibroblast differentiation, and collagen deposition. Thus, EMT has been implicated in the development of pulmonary fibrosis by generating collagen-producing myofibroblasts (12). Additionally, it was demonstrated that EMT plays a critical role in renal fibrosis. In the unilateral ureteral obstruction mice model, EMT-related protein Vimentin expression was required for renal fibrosis (13). The characteristics of EMT that are essential for wound healing also link EMT to interstitial fibrosis of the transplanted kidney. Our previous study established that EMT aggravated renal allograft interstitial fibrosis in CAD patients (14).

Organ fibrosis is a complex process involving numerous cytokines and growth factors. Among them, tumor necrosis factor-α (TNF-α) is first synthesized as transmembrane TNF-α, which is then cleaved by the TNF-α converting enzyme to release secretory TNF-α into the bloodstream and exert its effects (15). Several studies have already established a link between TNF-α and renal fibrosis; for example, TNF-α acted as a central mediator of a broad range of biological activities in kidney tubules, including cell proliferation, cell death, differentiation, as well as induction of inflammation and immune modulation, indicating that TNF-α was involved in obstructive kidney disease and diabetic nephropathy fibrosis (16). Our previous research established that TNF-α may activate EMT progression and that process was a critical mediator of renal graft interstitial fibrosis *via* regulation of ECM synthesis (14). Thus, inhibiting TNF-α-induced EMT could improve the survival of renal allografts.

Everolimus (EVR) is a pharmacological agent that was previously used to treat some types of solid tumors. EVR is approved by the Food and Drug Administration (FDA) for the treatment of metastatic renal cell carcinoma, advanced pancreatic neuroendocrine tumors, and advanced hormone receptor-positive breast cancer (17). EVR was recently reported to be used in some foreign clinical trials for anti-rejection therapy following kidney transplantation. According to the clinical study findings, EVR can significantly improve assessed glomerular filtration rate when compared to no change in the CsA group; however, it is associated with several adverse events (18, 19). In addition, a number of other pharmacologic actions of EVR have also been reported in some fibrotic diseases. According to the latest research, it has been reported that EVR attenuates tacrolimus-induced renal interstitial fibrosis in rats through suppressed TGF-β1 production (20). However, one research demonstrated that EVR led to serious pulmonary fibrosis and interstitial pneumonitis after administration in some cancer patients (21). Similarly, EVR also has the double face during the progression of EMT and fibrosis in in hepatic stellate cell and human liver cancer cells (22). Regrettably, the precise anti-fibrosis functional role of EVR in rat kidney transplant CAD model is not fully expounded. Previous studies always focused on the immunosuppression role of EVR. Therefore, a thorough examination of the underlying pharmacological mechanisms of EVR is critical for CAD prevention.

EVR, like Sirolimus and Tamsilorimus, exerts its anti-rejection effect by inhibiting the mammalian target of rapamycin (mTOR), a phosphoinositide 3-kinase-related protein that regulates cell cycle, protein synthesis, angiogenesis, and autophagy (23). Our research group previously revealed that ATG16L-dependent autophagic flux decreased gradually over time following kidney transplantation. In that study, we found that autophagy was downregulated in CAD patients and decreased LC3 predicted poor prognosis in CAD patients (24). Additionally, autophagy and EMT are two critical regulators of organ fibrosis progression and are closely related according to recent evidence (25). Just as autophagy plays a dual role in a variety of diseases, it also has a dichotomous effect in EMT, depending on the cell type and stage of fibrosis progression. Several investigations demonstrated that autophagy promoted EMT by upregulating the expression of an integrin-linked kinase, whereas autophagy enabled epithelial cells to acquire a mesenchymal phenotype (26). On the other hand, other results suggested that autophagy promoted TGF-β2-induced fibrosis by expressing the EMT phenotype during posterior capsular opacification (27). Therefore, the interaction between autophagy and EMT is complex and environment-specific.

In light of these findings, we postulated that EVR contributes to the prevention of interstitial fibrosis through modulating EMT. In this study, we revealed that EVR alleviated the progression of EMT and improved autophagic flux in kidney-transplanted rats. We then found that TNF-α-induced EMT was inhibited when the autophagy level was altered using EVR. Additionally, we investigated autophagy-independent potential underlying mechanisms of EVR using RNA sequencing. We found that the nuclear factor-κB (NF-κB) pathway was involved in TNF-α-induced EMT and that EVR could reverse this pathway activation by specifically stabilizing IκB-α.

## Results

### EVR Prevented Allograft Renal Function Impairment and Renal Interstitial Fibrosis in CAD Rat Model

**Figure 1:**
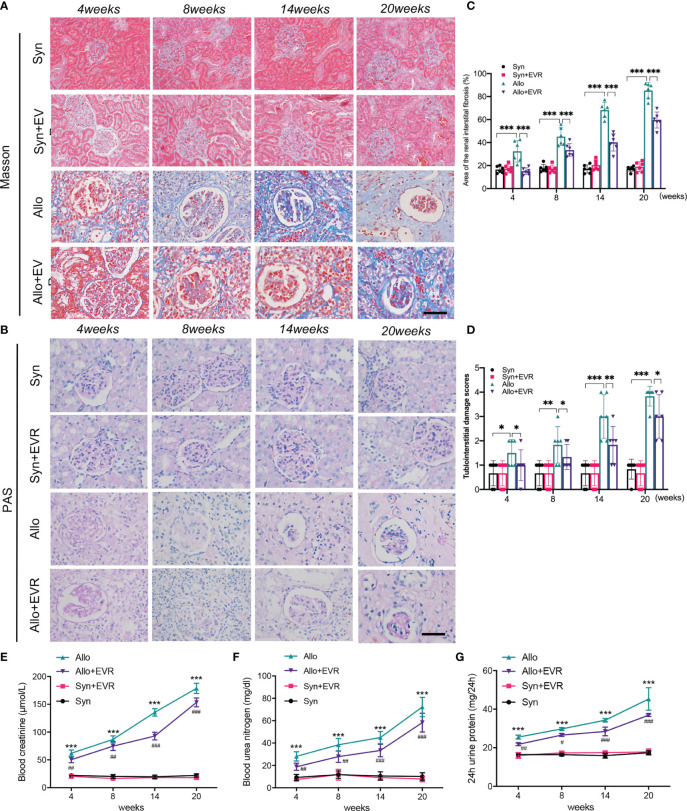
EVR prevented allograft renal function impairment and renal interstitial fibrosis in CAD rat model. **(A, B)** Representative images (*n* = 6) of Masson’s trichrome staining and PAS staining (scale bar: 25 μm) from kidneys of different treatment group rats. **(C)** Semi-quantitative analyses results of Masson’s trichrome staining positive area that represented the area of the renal interstitial fibrosis from kidneys of different treatment group rats. Values represented the mean ± SD (*n* = 6, ***p < 0.001). **(D)** The statistical analyses of tubulointerstitial damage scores in different treatment group rats. Values represented the mean ± SD (*n* = 6, *p < 0.05, **p < 0.01, ***p < 0.001). **(E–G)** Changes of renal function parameters—serum Cr, BUN, and 24-h urine protein of different treatment group rats. Values represented the mean ± SD (*n* = 6, ***p < 0.001 compared with control group, ^#^ *P* < 0.05, ^##^ *P* < 0.01, ^###^ *p* < 0.001 compared with Allo group); EVR, everolimus; PAS, periodic acid–Schiff; Cr, creatinine; BUN, blood urea nitrogen.

In order to explore the effect of EVR on renal allograft function and renal interstitial fibrosis progression in CAD rat model, we applied EVR (1.5 mg/kg, i.g., qod) to the syngeneic (syn) recipient rats and allogeneic (allo) recipient rats for different times. Masson’s trichrome staining and PAS staining assays were chosen to detect the pathological and renal allograft fibrosis level in each group of rats. Masson’s trichrome staining results of transplanted kidney samples showed that the Syn group had no renal interstitial fibrosis, while renal interstitial fibrosis was found in the Allo group at 4 weeks, and aggravated after 8 weeks. The results of Masson’s trichrome staining also showed that the blue-colored collagen fibers were gradually increased at 8, 14, and 20 weeks after kidney transplantation, compared with the Syn group. PAS staining assays of kidney tissue indicated varying degrees of glomerulosclerosis, tubular atrophy, and interstitial fibrosis in the Allo group at weeks 4, 8, 14, and 20 after kidney transplantation. The tubulointerstitial damage scores represented by PAS staining and the area of renal interstitial fibrosis represented by Masson staining were shown to indicate the development of CAD and renal interstitial fibrosis after renal transplantation in rats. However, treatment with EVR could obviously reduce structural damages and collagen deposition, as shown in PAS staining and Masson’s trichrome staining assays of transplanted kidneys (**Figures 1A–D**).

Serum creatinine (Scr), blood urea nitrogen (BUN), and 24-h urine protein are the most commonly used indicators of renal function. In order to test the effect of EVR on renal allograft function, the native right kidney from recipient rats was removed during kidney transplantation. The results showed that the renal allograft function started to be continuously deteriorated in the Allo group after the resection of right kidney compared with the Syn group. However, the increases of serum Cr, BUN, and 24-h urine protein in the Allo group were downregulated and they remained at a relatively low level when rats were treated by EVR at same time (**Figures 1E–G**).

### EVR Alleviated the Progression of EMT in CAD Rat Model

**Figure 2:**
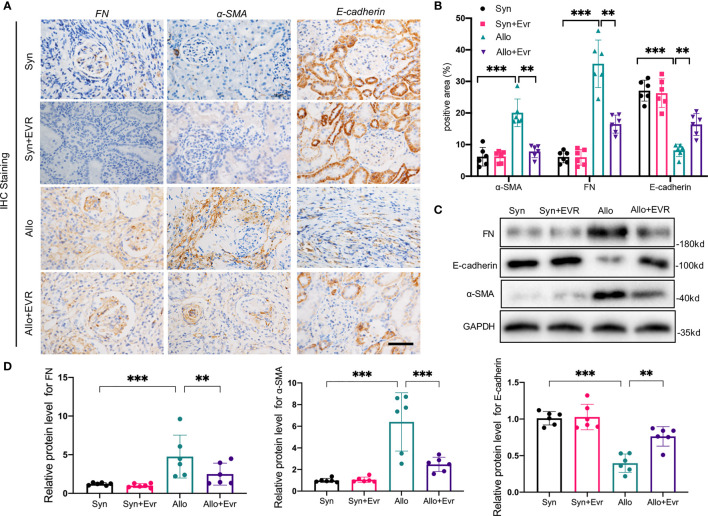
EVR alleviated the progression of renal tubular EMT in CAD rat model. **(A)** Representative IHC images of EMT markers (E-cadherin, α-SMA, and FN) in kidney sections from different treatment group rats (scale bar: 20 μm). **(B)** Semi-quantitative analyses results of IHC positive area in kidney sections from different treatment group rats. Values represented the mean ± SD (*n* = 6, **p < 0.01, ***p < 0.001). **(C)** Representative Western blotting results of EMT markers (E-cadherin, α-SMA, and FN) in kidney tissues from different treatment group rats. **(D)** Semi-quantitative analyses results of relative protein abundances of E-cadherin, α-SMA, and FN in kidney tissues from different treatment group rats. Values represented the mean ± SD (*n* = 6, **p < 0.01, ***p < 0.001); IHC, immunohistochemistry; α-SMA, α-smooth muscle actin; EVR, everolimus; EMT, epithelial–mesenchymal transition; FN, fibronectin.

In our previous study, we proved the increase of EMT in rat allograft kidney after kidney transplantation (24). Here, we explored the role of EVR in ameliorating the progression of renal allograft EMT after transplantation. EMT is characterized by cells that gradually lose epithelial cell markers, such as E-cadherin, and gain mesenchymal or myofibroblastic phenotype, such as α-smooth muscular actin (α-SMA) and fibronectin (FN). The results of immunohistochemistry (IHC) assays revealed the remarkably high expressions of α-SMA and FN and low expression of E-cadherin in the Allo group compared with the Syn group. However, the positive areas of α-SMA and FN in transplanted kidneys were remarkably decreased and the reduction of E-cadherin was relieved after the treatment of EVR in the Allo group (**Figures 2A, B**). Western blot (WB) analyses results also confirmed the outcomes of IHC assays (**Figures 2C, D**).

### EVR Activated Autophagy Flux in CAD Rat Model

**Figure 3:**
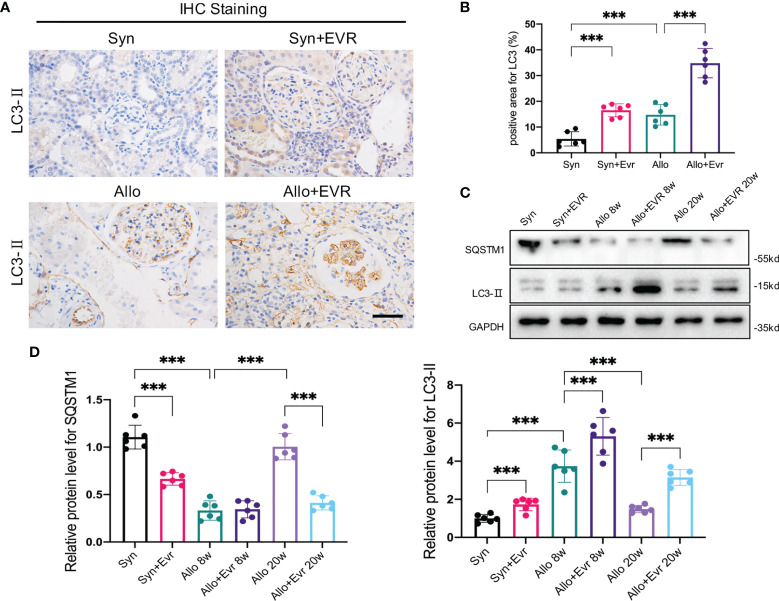
EVR activated autophagy in CAD rat model. **(A)** Representative IHC images of LC3-II in kidney sections from different treatment group rats (scale bar: 15 μm). **(B)** Semi-quantitative analyses results of IHC positive area in kidney sections from different treatment group rats. Values represented the mean ± SD (*n* = 6, ***p < 0.001). **(C)** Representative Western blotting results of autophagy markers (SQSTM1 and LC3-II) in kidney sections from different treatment group rats at 8 and 20 weeks. **(D)** Semi-quantitative analyses results of relative protein abundances of SQSTM1 and LC3-II in kidney sections from different treatment group rats at 8 and 20 weeks. Values represented the mean ± SD (*n* = 6, ***p < 0.001). EVR, everolimus.

The clinical utility of EVR is dependent on its ability to inhibit the mTOR pathway activation, which is frequently involved in protein synthesis, cell cycle and growth, angiogenesis, and autophagy. In our previous study, we discovered that autophagic flux generated by kidney transplantation gradually decreased over time (24). Therefore, we hypothesized that autophagy was involved in the effect of EVR on the progression of EMT and allograft renal interstitial fibrosis. Although IHC assays suggested that autophagy levels increased following kidney transplantation, EVR further up‐regulated LC3-II expression (**Figures 3A, B**). Again, WB assay results confirmed the accuracy of IHC assays and demonstrated that EVR could retard the impaired autophagic flux from 8 to 20 weeks (**Figures 3C, D**).

### EVR Activated Autophagic Flux Through the mTOR/ULK1 Pathway in HK2 Cells

**Figure 4:**
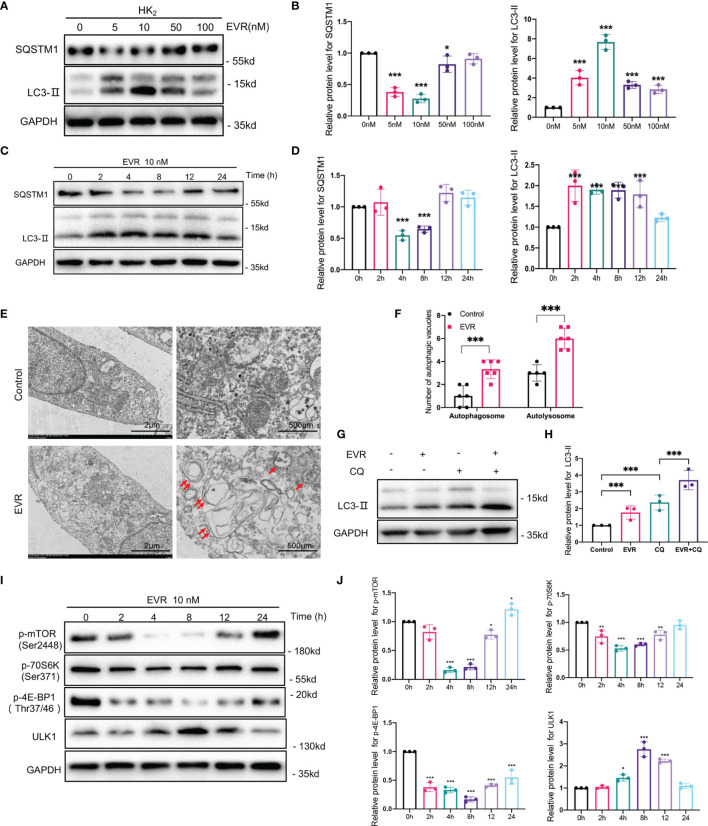
EVR activated autophagic flux through mTOR/ULK1 pathway in HK2 cells. **(A)** Representative Western blotting results of the expressions of SQSTM1 and LC3-II in HK2 cells after EVR treatment for different dosages. **(B)** Semi-quantitative analyses results of relative protein abundances of SQSTM1 and LC3-II in HK2 cells after EVR treatment for different dosage. Values represented the mean ± SD (*n* = 6, *p < 0.05, ***p < 0.001). **(C)** Representative Western blotting results of the expressions of SQSTM1 and LC3-II in HK2 cells after EVR treatment for different times. **(D)** Semi-quantitative analyses results of relative protein abundances of SQSTM1 and LC3-II in HK2 cells after EVR treatment for different times. Values represented the mean ± SD (*n* = 6, ***p < 0.001). **(E)** Representative TEM images of autophagosomes (red double arrow) and autolysosomes (red single arrow) in HK2 cells treated with or without EVR (10 nM) for 24 h (scale bar: 2 and 500 μm). **(F)** Qualitative analyses results of the number of autophagic vacuoles in control and EVR treatment groups under TEM. Values represented the mean ± SD (*n* = 6, ***p < 0.001). **(G)** Representative Western blotting results of the expression of LC3-II in HK2 cells treated with EVR (10 nM) and/or CQ (20 μM) for 24 h. **(H)** Semi-quantitative analyses results of relative protein abundance of LC3-II in HK2 cells treated with EVR and/or CQ for 24 h. Values represented the mean ± SD (*n* = 6, ***p < 0.001). **(I)** Representative Western blotting results of the expressions of p-mTOR, mTOR, p-70S6K, 70S6K, p-4E-BP1, 4E-BP1, and ULK1 in HK2 cells after EVR treatment for different times. **(J)** Semi-quantitative analyses results of relative protein abundances of p-mTOR, mTOR, p-70S6K, 70S6K, p-4E-BP1, 4E-BP1, and ULK1 in HK2 cells after EVR treatment for different times. Values represented the mean ± SD (*n* = 6, *p < 0.05, **p < 0.01, ***p < 0.001). TEM, transmission electron microscope; IF, immunofluorescence; EVR, everolimus; CQ, chloroquine.

We hypothesized that EVR might affect autophagy activity in HK2 cells. SQSTM1 is an autophagy substrate; its degradation and increase in MAP1LC3B-II (LC3-II) are markers of increased autophagy activity. WB analysis revealed that EVR increased LC3-II accumulation and decreased SQSTM1 in a dose- and time-dependent manner in HK2 cells. Autophagy level was found to be significantly increased following a 4-h exposure to EVR at a concentration of 10 nM (**Figures 4A–D**). The transmission electron microscope was used to demonstrate the typical organized cytoplasmic structure (autophagosome and autolysosome) formation. The autophagic process is completed when the autophagosome fuses with the lysosome to produce an autolysosome in which the substrates are degraded. As shown in **Figure 4E**, we found that EVR-treated HK2 cells had a greater number of autophagic vacuoles than the control group (**Figures 4E, F**). To further investigate EVR’s role in autophagic flux regulation, we then treated HK2 cells with EVR and chloroquine (CQ), simultaneously. CQ, a known inhibitor of autophagosome–lysosome fusion, results in increased autophagosomes, implying that autophagy is inhibited. When CQ inhibits autolysosome breakdown, the sustained increase in LC3 in response to stimulation indicates that the upstream of autophagy is activated. WB analysis revealed that when EVR was combined with CQ, the abundance of LC3-II and SQSTM1 was greatly increased in HK2 cells compared to when EVR was administered alone (**Figures 4G, H**). This illustrated that the role of EVR was to induce autophagy upstream.

mTOR signaling is critical for autophagy regulation, with activated mTOR suppressing autophagy, and blocking of mTOR inducing it. Additionally, as shown in **Figure 4E**, we used WB assays to detect total mTOR and mTOR phosphorylation (p-mTOR). As expected, EVR significantly blocked mTOR activity, as did phosphorylation of both 4E-BP1 and p70S6K, which are downstream mediators of mTOR in HK2 cells. Meanwhile, EVR-treated HK2 cells exhibited an increase in ULK1, one of the major downstream targets of the mTOR pathway involved in autophagy regulation. Taken together, these findings indicate that EVR can significantly increase the autophagic flux in HK2 cells *via* the mTOR/ULK1 pathway (**Figures 4I, J**).

### EVR Alleviated TNF-α‐Mediated EMT, Cell Migration, and Invasion in HK2 Cells

**Figure 5:**
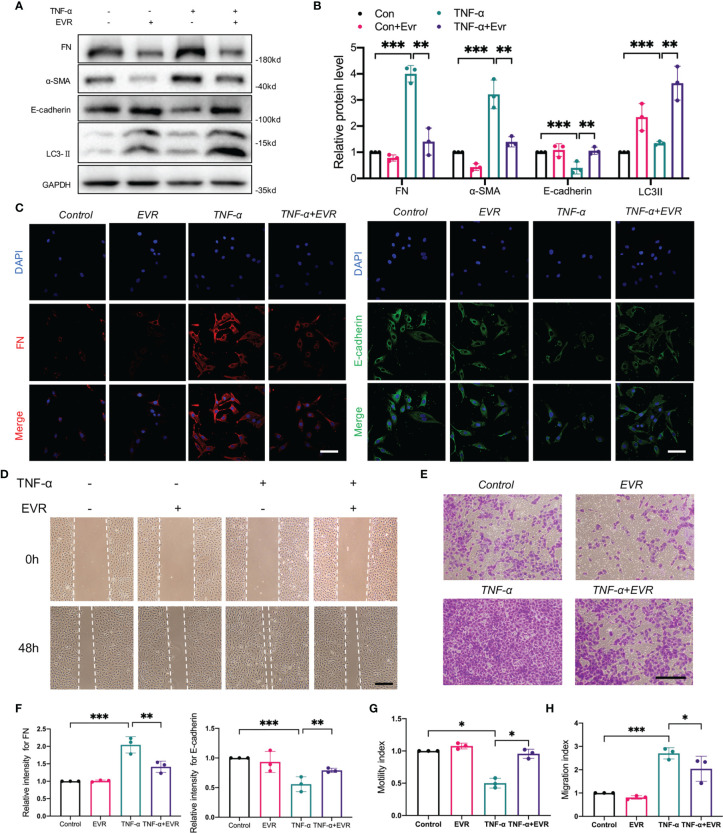
EVR alleviated the TNF-α‐mediated EMT, cell migration, and invasion in HK2 cells. **(A)** Representative Western blotting results of EMT markers (E-cadherin, α-SMA, and FN) and LC3-II in HK-2 cells treated with TNF-α (50 ng/ml) and/or EVR (10 nM) for 48 h. **(B)** Semi-quantitative analyses results of relative protein abundances of E-cadherin, α-SMA, FN, and LC3-II in HK-2 cells treated with TNF-α and/or EVR for 48 h. Values represented the mean ± SD (*n* = 6, **p < 0.01, ***p < 0.001). **(C)** Representative IF staining images of E-cadherin and FN in HK-2 cells treated with TNF-α (50 ng/ml) and/or EVR (10 nM) for 24 h (scale bar: 20 μm). **(D)** Representative wound healing test images on HK-2 cells treated with TNF-α (50 ng/ml) and/or EVR (10 nM) for 48 h (scale bar: 20 μm). **(E)** Representative transwell assay images on HK-2 cells treated with TNF-α (50 ng/ml) and/or EVR (10 nM) for 48 h (scale bar: 20 μm). **(F)** Semi-quantitative analyses results of relative IF staining intensity of E-cadherin and FN in HK-2 cells treated with TNF-α (50 ng/ml) and/or EVR (10 nM) for 24 h. Values represented the mean ± SD (*n* = 3, **p < 0.01, ***p < 0.001). **(G)** Quantitative analyses results of the motility index of HK-2 cells treated with TNF-α and/or EVR for 48 h; the motility index has determined by the formula “migration cell number of control group/migration cell number of the other treatment group”. Values represented the mean ± SD (*n* = 3, *p < 0.05). **(H)** Quantitative analyses results of the migration index of HK-2 cells treated with TNF-α and/or EVR for 48 h; the migration index as determined by the formula “migration cell number of the other treatment group/migration cell number of control group”. Values represented the mean ± SD (*n* = 3, *p < 0.05, ***p < 0.001). α-SMA, α-smooth muscle actin; EVR, everolimus; EMT, epithelial–mesenchymal transition; FN, fibronectin; TNF-α, tumor necrosis factor-α.

Our previous research established that TNF-α-induced EMT plays an important role in the progression of allograft kidney fibrosis (28). HK2 cells were treated with 50 ng/ml TNF-α with or without EVR to mimic the *in vivo* condition. EVR significantly decreased the protein expressions of α‐SMA and FN induced by TNF-α, while partially restoring the expression of E‐cadherin (**Figures 5A, B**). Additionally, the results of IF staining for FN and E-cadherin revealed that EVR can inhibit the EMT progression of HK2 cells generated by TNF-α (**Figure 5C**).

Additionally, we assessed changes in cell migration and invasion ability, which are highly associated with EMT. The wound healing test and Transwell assays were used to assess the acquisition of migratory and invasive capacity following treatment with TNF-α and EVR, or in their absence. TNF-α significantly increased the chemokinetic motility of HK2 cells in wound healing experiments, an effect that was reversed by EVR. EVR, similarly, significantly inhibited the TNF-α-induced chemotactic response and migration (**Figures 5D–H**). These findings supported the hypothesis that treating HK-2 cells with EVR could ameliorate TNF-induced EMT.

### Blockade of Autophagy Promoted TNF-α‐Mediated EMT and Diminished Action of EVR on This Process

To further demonstrate the involvement of autophagy in EVR inhibition of TNF-α‐meditated EMT *in vitro*, we first examined the protein expression of α‐SMA, E‐cadherin, and FN in HK2 cells treated with various concentrations of TNF-α and CQ. The WB results demonstrated that CQ at 20 and 40 µM significantly promoted the progression of TNF-α‐induced EMT (**Supplementary Figure 1A**). Meanwhile, we found that EVR was capable of decreasing α‐SMA and FN expression while increasing E‐cadherin expression in the TNF-α+EVR group compared to the TNF-α group. Additionally, the progression of EMT was aggravated in the TNF-α+EVR+CQ group compared to the TNF-α+EVR group, and it was almost recovered with the same level of treatment with TNF-α alone (**Supplementary Figure 1B**). These changes in EMT marker expression were accentuated by co-stimulation with TNF-α and CQ, and the protective effect of EVR against TNF-α‐induced EMT could be recovered if CQ was used to block autophagy.

### EVR Suppressed TNF-α-Mediated EMT *via* Blocking NF-κB Instead of Akt Signaling Pathway in HK-2 Cells

**Figure 6:**
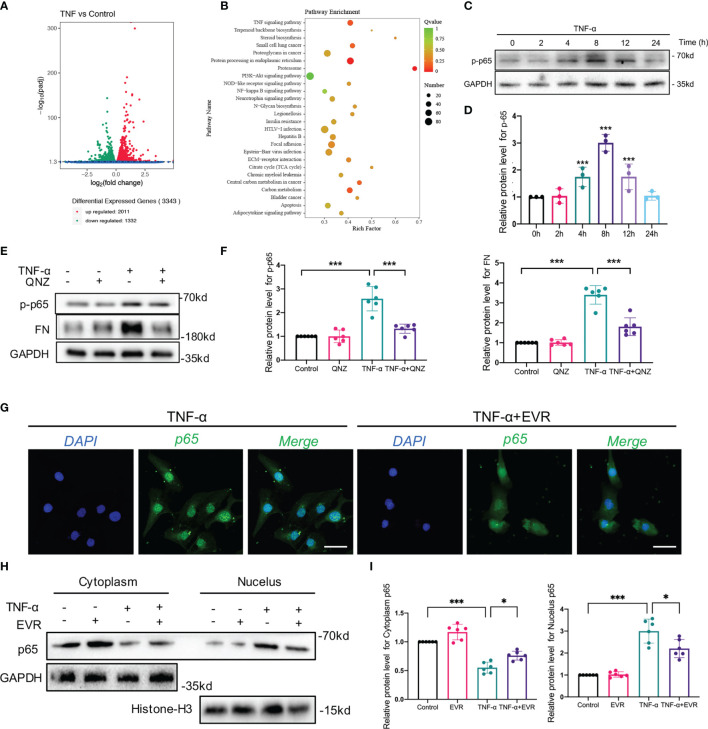
EVR suppressed TNF-α-mediated EMT *via* blocking NF–κB instead of Akt signaling pathway in HK-2 cells. **(A)** The RNA sequencing results of differential genes in HK-2 cells treated with or without TNF-α (50 ng/ml) for 24 h (*n* = 5). **(B)** KEGG pathway analyses results of the top 20 KEGG enriched gene pathways base on RNA sequencing results. **(C)** Representative Western blotting results of the expression of p-p65 in HK2 cells after TNF-α (50 ng/ml) treatment for different times. **(D)** Semi-quantitative analyses results of relative protein abundance of p-p65 in HK2 cells after TNF-α treatment for different times. Values represented the mean ± SD (*n* = 6, ***p < 0.001). **(E)** Representative Western blotting results of the expressions of p-p65 and FN in HK2 cells treated with TNF-α (50 ng/ml) and/or QNZ (20 nM) for 24 h. **(F)** Semi-quantitative analyses results of relative protein abundances of p-p65 and FN in HK2 cells treated with TNF-α and/or QNZ for 24 h. Values represented the mean ± SD (*n* = 6, ***p < 0.001). **(G)** Representative IF staining images of p65 in HK-2 cells treated with TNF-α (50 ng/ml) and/or EVR (10 nM) for 24 h (scale bar: 50 μm). **(H)** Representative Western blotting results of p65 in cytoplasmic and nuclear fractions of HK-2 cells treated with TNF-α (50 ng/ml) and/or EVR (10 nM) for 24 h. **(I)** Semi-quantitative analyses results of relative protein abundance of p65 in HK-2 cells treated with TNF-α and/or EVR for 24 h. Values represented the mean ± SD (*n* = 6, *p < 0.05, ***p < 0.001). TNF-α, tumor necrosis factor-α; KEGG, Kyoto Encyclopedia of Genes and Genomes; FN, fibronectin; QNZ, NF−κB inhibitor quinazoline; IF, immunofluorescence; EVR, everolimus.

To further elucidate mechanisms of EVR on TNF-α-induced EMT, besides autophagy, we first performed whole-transcriptome RNA sequencing (RNA-seq) on HK2 cells treated with or without TNF-α. A comparison of the TNF-α group and control groups revealed that 2011 differentially expressed genes were highly expressed while 1332 genes showed decreased expression (**Figure 6A**). The number of downregulated differentially expression gene with log fold change (FC) value lower than minus 2 and *p*-value less than 0.05 is 531. The number of upregulated differentially expression gene with logFC value higher than 2 and *p*-value less than 0.05 is 355.

KEGG pathway analysis of the RNA-seq data revealed that a total of 167 pathways were identified, 26 of which were significantly enriched in the TNF-α group compared to the control group. Top pathways identified by KEGG analysis ranked by *p*-values (**Figure 6B**). In our previous study, we demonstrated that Akt and NF-κB signaling pathway activation was induced by TNF-α in HK2 cells. In our KEGG pathway analysis, the input gene numbers of Akt and the NF-κB signaling pathway are 82 and 28, respectively (*p*-value < 0.001). To corroborate the RNA-seq data, we used WB analysis to determine whether TNF-α activated the Akt signaling pathway in a time-dependent manner (**Supplementary Figures 2A, B**). To investigate the involvement of the Akt pathway in TNF-α-induced EMT, we suppressed Akt phosphorylation in HK2 cells using MK2206, an Akt pathway-specific inhibitor, and observed that MK2206 significantly decreased FN expression induced by TNF-α (**Supplementary Figures 2C, D**).

When we investigated the effect of EVR on the TNF-activated-Akt pathway, however, we found that the TNF-activated Akt pathway was not significantly inhibited by EVR intervention (**Supplementary Figures 2E, F**). This phenomenon could be explained by the fact that EVR acts as an inhibitor of mTOR, whereas Akt is the upstream effector of the mTOR pathway.

To investigate a second latent mechanism through which EVR inhibits TNF-α-induced EMT, we identified many important mediators and their phosphorylation levels in the NF-κB pathway, which was also significantly enriched by the KEGG pathway analysis. The results confirmed our expectations that TNF-α may promote phospho-p65 (p-p65), a marker of the NF-κB pathway activation, in a time-dependent manner (**Figures 6C, D**). TNF-α-induced EMT progression was reversed following treatment with QNZ, a specific inhibitor of the NF-κB pathway that inhibited p-p65 expression (**Figures 6E, F**). Given that p-p65 has been shown to activate the NF-κB pathway by promoting p65 nuclear translocation, we also investigated the effect of EVR on p65 nuclear translocation. The results of IF staining indicated that TNF-α promoted p65 nuclear translocation while increasing cytosolic p65 increased and decreasing nuclear p65 following the addition of EVR in TNF-α-treated HK2 cells (**Figure 6G**). Consistent with the IF staining results, the WB analysis of p65 protein expression in the nucleus and cytoplasm yielded the same results (**Figures 6H, I**).

### EVR Inhibited TNF-α-Mediated EMT by Inhibiting Degradation of IκB-α in HK-2 Cells, and Skp2 Played an Essential Role in This Process

**Figure 7:**
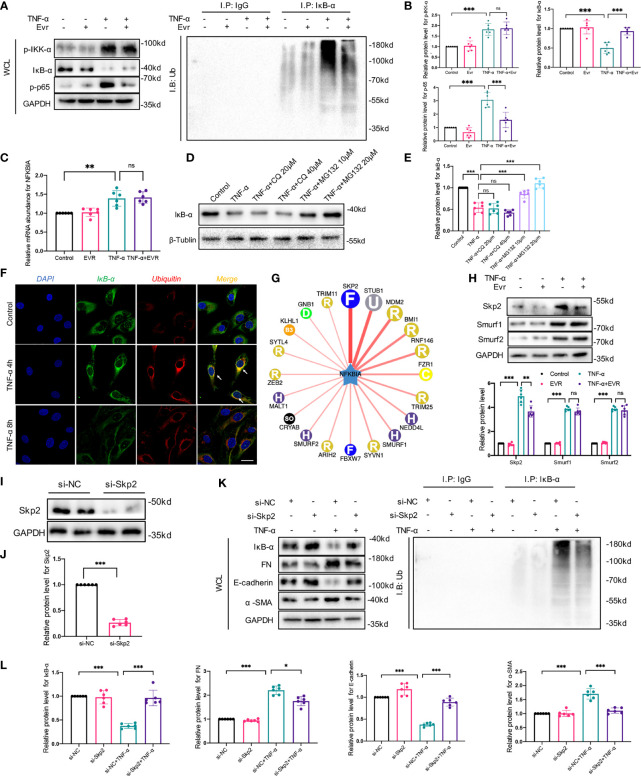
EVR inhibited TNF-α-induced EMT by inhibiting degradation of IκB-α in HK-2 cells, and Skp2 played an essential role in this process. **(A)** Representative Western blotting results f p-IKK-α, IKK-α, IκB-α, p-p65, and p65 in WCL of HK-2 cells treated with TNF-α (50 ng/ml) and/or EVR (10 nM) for 24 h. In addition, immunoprecipitation was performed with a IκB-α antibody, and Western blot assays were used to examine the ubiquitin conjugation among the indicated groups. **(B)** Semi-quantitative analyses results of relative protein abundances of p-IKK-α, IKK-α, IκB-α, p-p65, and p65 in WCL of HK-2 cells treated with TNF-α and/or EVR for 24 h. Values represented the mean ± SD (*n* = 6, ns, not significant, ***p < 0.001). **(C)** Relative expression of the NFKBIA in HK-2 cells treated with TNF-α (50 ng/ml) and/or EVR (10 nM) for 24 h. Values represented the mean ± SD (*n* = 6, ns, not significant, **p < 0.01). **(D)** Representative Western blotting results of IκB-α in HK2 cells treated with TNF-α (50 ng/ml) and different concentrations of CQ (20 and 40 μM)/MG132 (10 and 20 μM) for 24 h. **(E)** Semi-quantitative analyses results of relative protein abundance of IκB-α in HK-2 cells treated with TNF-α and CQ/MG132 for 24 h. Values represented the mean ± SD (*n* = 6, ns, not significant, ***p < 0.001). **(F)** Representative IF co-localization staining images of IκB-α and ubiquitin in HK-2 cells treated with TNF-α for 4 or 8 h (scale bar: 50 μm). **(G)** The network map of E3 ubiquitin ligase related to IκB-α predicted by UbiBrowser. **(H)** Representative Western blotting results of Skp2, Smurf1, and Smurf2 in HK2 cells treated with TNF-α (50 ng/ml) and/or EVR (10 nM) for 24 h. **(I, J)** Representative Western blotting results of negative control (NC) and small interfering RNA (si-Skp2) transfection efficiency in HK2 cells. **(K, L)** Representative Western blotting results of IκB-α, E-cadherin, α-SMA and FN in WCL of HK-2 cells treated with TNF-α (50 ng/ml) after transfecting si-Skp2 or si-NC. Immunoprecipitation was also performed with an IκB-α antibody, and Western blot assays were used to examine the ubiquitin conjugation among the indicated groups. Values represented the mean ± SD (*n* = 6, *p < 0.05, ***p < 0.001). WCL, whole-cell lysates; IF, immunofluorescence; EVR, everolimus; CQ, chloroquine; α-SMA, α-smooth muscle actin; FN, fibronectin.

Given that phosphorylation/degradation of IκB-α precedes nuclear translocation of NF-κB and that upstream mediator IKK activates phosphorylation/degradation of IκB-α, we investigated IκB-α and IKK phosphorylation to elucidate the inhibitory effect of EVR on the upstream event of NF-κB. Then, using a Western blot assay, we identified several key downstream mediators of the NF-κB pathway in whole-cell lysates (WCL). We found that EVR significantly upregulated IκB-α and downregulated p-p65 protein in the presence of TNF-α stimulation, whereas total p65, IKK-α, and their protein phosphorylation levels remained unchanged compared to the TNF-α-treated group (**Figure 7A**). To ascertain if IκB-α degradation was responsible for NF-κB pathway activation, we also examined the relationship between IκB-α and ubiquitination. We found that IκB-α was coprecipitated with ubiquitin following TNF-α treatment and that this trend could be reversed by EVR (**Figures 7A, B**). Next, we determined whether TNF-α treatment-induced IκB-α downregulation was due to increased degradation or decreased synthesis at the transcriptional level. In contrast to IκB-α protein, NFKBIA mRNA (encoding IκB-α) was increased in TNF-α-treated cells and EVR pretreatment has no effect on this response (**Figure 7C**). This revealed that the EVR-mediated upregulation of IκB-α may be related to increased protein stability rather than increased mRNA expression. Therefore, we identified the roles of two major pathways—autophagy and ubiquitination, which are thought to be responsible for protein degradation on IκB-α protein levels. With TNF-α treatment, the presence of MG132, a ubiquitin–proteasome-mediated degradation inhibitor, significantly increased the stability of IκB-α protein. However, there was no significant variation in IκB-α protein levels when different concentrations of CQ were used (**Figures 7D, E**). The IF co-staining for ubiquitination (red immunofluorescence) and IκB-α (green immunofluorescence) also indicated that the ubiquitin–proteasome pathway was primarily responsible for IκB-α degradation (**Figure 7F**).

To elucidate the mechanism by which EVR inhibited IκB-α ubiquitination and degradation, we predicted the E3 ubiquitin ligase of IκB-α using UbiBrowser, an integrated bioinformatics platform (http://ubibrowser.ncpsb.org) (**Figure 7G**). According to our predicted results, we screened 3 genes (Skp2, Smurf1, and Smurf2) that were upregulated by TNF-α. Among these, EVR significantly decreased the Skp2 protein expression (**Figure 7H**). To further elucidate the function of Skp2 on IκB-α, Skp2 was knocked down using small interfering RNA (siRNA) (**Figures 7I, J**). As demonstrated in our previous study, si-Skp2 functions were similar to EVR. Knockdown of Skp2 expression decreased EMT progression and attenuated the IκB-α decrease in TNF-α-treated HK2 cells (**Figures 7K, L**).

### EVR Upregulated IκB-α Protein and Downregulated Skp2 and p-p65 in CAD Rat Model

**Figure 8:**
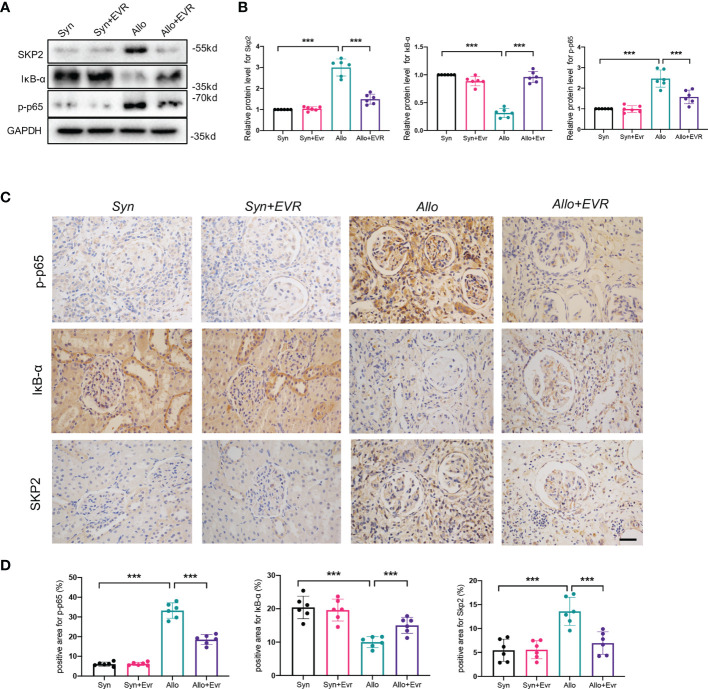
EVR upregulated IκB-α protein and downregulated Skp2 and p-p65 in the CAD rat model. **(A)** Representative Western blotting results of Skp2, IκB-α, and p-p65 in kidney tissues from different treatment group rats. **(B)** Semi-quantitative analyses results of relative protein abundances of Skp2, IκB-α, and p-p65 in kidney tissues from different treatment group rats. Values represented the mean ± SD (*n* = 6, ***p < 0.001). **(C)** Representative IHC images of Skp2, IκB-α, and p-p65 in kidney sections from different treatment group rats (scale bar: 10 μm). **(D)** Semi-quantitative analyses results of IHC positive staining of Skp2, IκB-α, and p-p65 in kidney sections from different treatment group rats. Values represented the mean ± SD (*n* = 6, ***p < 0.001).

We used WB and IHC staining assays to confirm that IκB-α protein plays an essential role in the EVR-induced EMT inhibition in rat allograft kidneys. The results of WB demonstrated that the allo group expressed remarkably high levels of Skp2 and p-p65, and low expression of IκB-α, whereas EVR was able to reverse these proteins’ expressions (**Figures 8A, B**). Additionally, the IHC staining experiment corroborated the results of the Western blot assay (**Figures 8C, D**). These findings suggested that the mechanism by which EMT progresses in CAD allografts may be related to dysregulation of IκB-α protein degradation and that EVR may play a role in stabilizing IκB-α protein by inhibiting Skp2.

## Discussion

**Figure 9:**
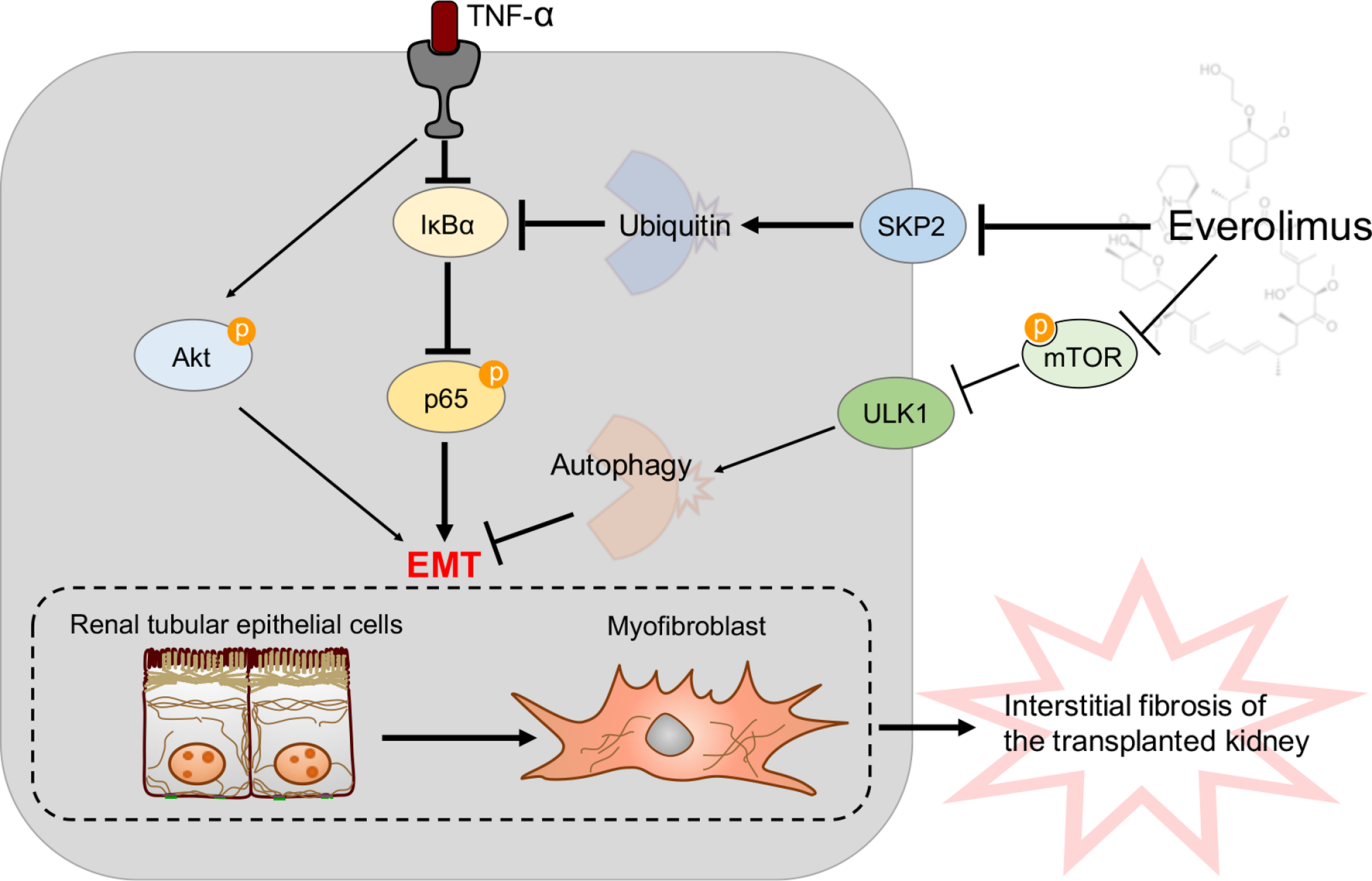
A model is proposed to illustrate the protective mechanisms of EVR on the pathogenesis of transplanted renal tubular EMT and interstitial fibrosis in CAD. TNF-α could induce renal tubular EMT and then promote transplanted renal interstitial fibrosis through Akt and NF-κB signaling pathway activation in CAD recipients. Of these, NF-κB signaling pathway activation was due to IκB-α decrease and p-p65 increase. On the one hand, EVR could inhibit progression of renal tubular EMT *via* blocking the mTOR/ULK1 pathway to activate autophagy flux directly. On the other hand, EVR could also affect Skp2 (a E3 ubiquitin ligase) activity and inhibit IκB-α ubiquitin degradation, and thus restrain progression of renal tubular EMT through impacting NF-κB pathway activation. Red arrows represent the effect of TNF-α and dark blue arrows represent the effect of EVR.

In the present study, we demonstrated that EVR inhibited the TNF-α-induced EMT by activating autophagy and stabilizing IκB-α protein degradation, thus attenuating interstitial fibrosis in transplanted kidneys (**Figure 9**). TNF-α promotes EMT in HK2 cells by activating both the Akt and NF-κB pathways. However, it appeared as though EVR had an effect on NF-κB activation rather than the Akt signaling pathway. Although EVR is considered an immunosuppressive agent, it is only used in a few countries in the clinical treatment of rejection reactions. Since EVR can inhibit the mTOR pathway, it is able to regulate autophagy. However, a limited number of studies have been reported that show that EVR influences organ fibrosis through autophagy. Only one reported that EVR reduces postoperative arthrofibrosis inducing autophagy-mediated fibroblast apoptosis (29). To the best of our knowledge, this is the first study to examine the effects and underlying mechanism of EVR on renal interstitial fibrosis in CAD recipients to prevent impaired autophagic flux. Our findings provide new insights into allograft renal interstitial fibrosis treatment and prevention. Additionally, our findings revealed additional novel insights into EVR pharmacological targets that may facilitate CAD modulation. A pharmaceutical intervention that inhibits autophagy, such as EVR, is a promising first-line treatment option for patients who have undergone kidney transplantation.

Although CAD manifests in a variety of ways and causes a variety of different forms of kidney injury, the underlying molecular process remains unknown. EMT is the initial stage of irreversible tubular injury and interstitial fibrosis, which is followed by increasing loss of renal function and eventually results in an annual loss of approximately 5% of allograft function. Unfortunately, while numerous studies demonstrated that EMT was a classic source of myofibroblasts and fibroblasts during renal fibrosis, few effective clinical therapeutic strategies for reversing the course of these modifications were discovered. We previously demonstrated that TNF-α promoted EMT in HK2 cells by activating the Akt pathway, and this process may play a critical role in exacerbating interstitial fibrosis in CAD patients with transplanted kidneys (28). Therefore, in this study, we sought to discover a novel therapeutic agent capable of reversing the course of CAD by inhibiting TNF-α-induced EMT.

Recently, due to its immunosuppressive effect, EVR has garnered much greater attention as a molecular targeting drug (18). Meanwhile, EVR has anti-pulmonary fibrosis, anti-renal fibrosis, anti-arthrofibrosis, and anti-hepatic fibrosis therapeutic potential in addition to its widespread use in traditional anti-cancer therapies (21, 29, 30). However, overall safety data for EVR in CAD patients, obtained from controlled research with the CsA group, revealed that EVR was superior to measured glomerular filtration rate (mGFR), but was associated with more adverse events (19). This study suggested that poor tolerance to EVR was closely related to its dose-limiting toxicity (19). Therefore, we investigated the dose-dependent effect of EVR on HK2 cells treated with different concentrations of EVR, i.e., 5, 10, 50, and 100 nM for 24 h. We discovered that autophagy levels were much lower in the high-dose EVR-treated group than in the control group. Therefore, when we performed other interventions with cells in this study, the concentration of EVR was strictly controlled. The findings were consistent with those from previous studies that high-dose EVR (at least more than 100 nM) could induce EMT in airway epithelial cells and exacerbate pulmonary fibrosis (31). Furthermore, other investigations revealed that the results in intact animals were consistent with the *in vitro* findings that high-dose EVR induced worsening of renal function, but that early treatment with a low dose of EVR was effective in preventing rat kidney injury (32). According to some reports, we assumed that this side effect of EVR was caused by excessive autophagic activation (33, 34).

We previously established that ATG16L and autophagic flux were activated in the early stages of kidney transplantation, but that autophagic flux reduced in a dynamic range at a late stage. mTOR signaling inhibition has been shown to have a positive effect on the cellular autophagy cascade (33, 35). On this basis, we hypothesized that EVR exerted its anti‐fibrosis effects through the resistance of impaired autophagic flux following kidney transplantation. To investigate the association between EVR, autophagy, and TNF-α-induced EMT in the transplanted kidneys, EVR was used at an optimum selection concentration in the CAD rat model and HK2 cells. We explored whether EVR could help alleviate renal allograft interstitial fibrosis and the progression of EMT in CAD rats. Meanwhile, autophagy was increased in the Allo+EVR group compared to rats that had only received transplantation, and EVR aided in maintaining a stable state of autophagy flux. These findings were also confirmed in HK2 cells, where EVR restored epithelial cell markers and decreased mesenchymal marker expressions in TNF-α-treated HK2 cells *via* autophagy activation. However, our findings appeared to contradict the findings of Nakagawa et al., that EVR-induced autophagy aggravates tubular dysfunction during kidney injury recovery (34). We hypothesized that the mechanism of action would differ according to the role of autophagy in different pathological environments.

To gain additional insights into the potential mechanisms of EVR on TNF-α-mediated-EMT, total RNA from HK2 cells treated with TNF-α and control cultures were sequenced by RNA-seq. KEGG pathway analysis revealed a significant enrichment of the Akt pathway, which is well known to regulate mTOR upstream. Therefore, we first assumed that EVR had a direct effect on TNF-α-mediated EMT *via* the Akt pathway. Contrary to expectations, we discovered that TNF-α could activate Akt but EVR did not affect Akt phosphorylation. However, other investigations revealed that EVR could reduce arthrofibrosis by downregulating the expression levels of p-PI3K, p-Akt, and p-mTOR (29). Additionally, RNA-seq data revealed a link between EVR and TNF-α-mediated EMT in HK2 cells *via* the NF-κB pathway.

Members of the IκB family include NF-κB1 (p105), NF-κB2 (p100), IκB-α, IκB-β, and IκB-ϵ. Among the IκB family proteins, IκB-α mainly mediates basal inhibition of NF-κB activity. The activated IκB kinase complex phosphorylates IκB-α, which is then followed by the ubiquitination of IκB-α and its subsequent degradation by the proteosome. External stimulatory signals first activate the IκB kinase (IKK), which subsequently phosphorylates IκB-α and decomposes the NF-κB–IκB complex. This process is a prerequisite for NF-κB pathway activation, with p-p65 nuclear translocation being the outcome (36). Therefore, we examined the effect of EVR on the expression of IKK, p-IKK, IκB-α, p65, and p-p65 proteins following TNF-α and/or EVR treatment. In general, p-IKK-mediated IκB-α phosphorylation resulted in its ubiquitination and subsequent degradation (36). Nevertheless, several investigations demonstrated that TNF-α promoted biphasic degradation of IκB-α in intestinal epithelial cells *via* autophagy and the proteasome (37). However, our data indicated that autophagy played a lesser role in the stabilization of IκB-α in epithelial cells of the renal tubules. According to the WB analysis results, EVR had no effect on TNF-α-activated IKK phosphorylation but inhibited IκB-α ubiquitylation degradation. Next, we performed a predictive screening of E3 ubiquitin ligases known to affect IκB-α degradation and found that EVR could decrease IκB-α degradation by inhibiting Skp2 expression. The relationship between rapamycin and Skp2 has been previously described in breast cancer (38). Coincidentally, it has been demonstrated that EVR leads to a suppression of their paracrine and accelerate osteoclast activity by interfering with the NF-κB pathway (39). However, there is no in-depth research to explain how EVR affects this pathway. Our research suggests that derivatives of rapamycin-EVR may also prevent TNF-α-induced EMT by downregulating Skp2 to inhibit IκB-α ubiquitination degradation and NF-κB pathway activation.

Our research has several limitations. The current validation of EVR’s effects on TNF-α-induced EMT and renal allograft interstitial fibrosis was conducted in an epithelial cell line and animal model, which may not accurately mimic the complexity of *in vivo* changes occurring during CAD progression. Although we successfully established a rat CAD model to investigate the effects of EVR on the establishment of renal tubular EMT and interstitial fibrosis in allograft kidneys, further confirmation of the role of NF-κB signaling pathway and IκB-α protein on this process may require using knockout mice. Additionally, data from patients treated with EVR after kidney transplantation are also important. At the time of reporting, no therapy with EVR had been attempted in our patients. EVR’s safety and long-term effects as an immunosuppressant must still be explored further before it is approved for clinical use.

The findings presented in this paper demonstrate that EVR can attenuate TNF-α-induced EMT and interstitial fibrosis in renal allografts. Our findings further demonstrate that EVR’s therapeutic potential is not limited to its ability to induce autophagy, but also to its effect on NF-κB signaling inactivity by inhibiting IκB-α protein ubiquitylation degradation, and Skp2 may serve as a novel target protein between EVR and IκB-α. The findings of this study provide novel insight into the role of EVR in the autophagy-dependent and autophagy-independent pathways. The development of more autophagy-targeting drugs for the treatment and prevention of renal interstitial fibrosis and CAD progression in kidney transplant recipients is expected in the near future. Altogether, the present investigation will contribute to the development of new clinical interventions for CAD patients, and EVR may be recommended as a novel option for the prevention and treatment of renal allograft interstitial fibrosis, in addition to its immunosuppressive effects.

## Materials and Methods

### Rats and Animal Models

First, to minimize alloimmunity, isogeneic transplants were performed between inbred rats. Based on our pervious observations (28) and on the literature (40, 41), a classical rat model of kidney transplantation was established. The stability of the genetic background in inbred rats avoided the fluctuation in the parameters. Thus, adult male Lewis (LEW/Crl; LEW) inbred rats and F344 (F344/DuCrl; CDF) inbred rats (Age 6 to 8 weeks, weight 250 ± 10.3 g) were purchased from Charles River Laboratories (Beijing, China). To observe ethical codes of research, approval of Institutional Animal Care and Use Committee at Nanjing Medical University was obtained (ID: IACUC-2010020). Animal handling was done adhering to the guidelines for animal handling at the Nanjing Medical University and the guidelines published by the US National Institutes of Health. The place for animal experiments was the Laboratory Animal Center of Nanjing Medical University.

Orthotopic left kidney transplantation was performed between F344 and Lewis rats as previously described (28). Right nephrectomy was performed simultaneously. The cold ischemia time was defined as the grafts were stored in cold saline and the warm ischemia time was defined as the period between the cessation of donor blood supply to an organ and the beginning of cold perfusion. Mean cold ischemia time for the kidney transplants was less than 30 min, and for the warm ischemia, it was less than 10 s. A low dose of cyclosporine A (5 mg/kg, qd, ip; Neoral, Novartis, Switzerland) was administered for 10 days after kidney transplantation surgery to prevent acute rejection.

Rats were randomly divided into four groups of 6 rats each before kidney transplantation: Lewis rats used as syngeneic donors were defined as the Syn group (Lewis to Lewis), Lewis rats used as recipients were defined as the Allo group (F344 to Lewis), and the Syn and Allo group rats treated with EVR (1.5 mg/kg body weight, Novartis, Basel, Switzerland) were defined as the Syn+EVR group and the Allo+EVR group. Both groups were treated with EVR or a matched vehicle by oral gavage once per 2 days, continuing for 8 to 20 weeks. Kidneys, serum, and urine samples were collected at 8, 12, 16, and 20 weeks after kidney transplantation. The levels of blood urea nitrogen and serum creatinine were measured to examine renal function by using rat QuantiChromassay kits (Jiancheng, Beijing, China). The 24-h urinary protein concentration was determined by the commercial kit (Mlbio, Shanghai, China) according to the instructions of the manufacturer.

### Renal Histological Pathology Analyses

Histological analyses were performed by using periodic acid–Schiff (PAS) and Masson’s trichome staining. PAS and Masson’s trichome staining were performed as previously described (28), which was used to evaluate the severity of tubular injury (tubular lumen dilation, cast formation, tubular cell sloughing, atrophy, and basement membrane thickening) and the area of renal interstitial fibrosis separately. Two pathologists double-blindly read the pathological sections and used Image‐Pro Plus (Media Cybernetics, Rockville, MD) to quantify the morphometric changes of the transplanted kidneys.

### Immunohistochemistry Staining Assay

Kidney samples were fixed in 10% neutral formalin and embedded in paraffin. Three-micrometer-thick sections were used for immunohistochemical staining assay. Simply, paraffin-embedded kidney sections were deparaffinized, hydrated, and antigen-retrieved, and endogenous peroxidase activity was quenched by 3% H_2_O_2_. Sections were then blocked with 10% normal donkey serum, followed by incubation with anti-FN (Abcam, USA), anti-α-SMA (Abcam, USA), anti-E-cadherin (Abcam, USA), anti-LC3 (CST, USA), anti-p-p65 (CST, USA), and anti-Skp2 (CST, USA) overnight at 4°C. After incubation with secondary antibody for 1 h, sections were incubated with ABC reagents for 1 h at room temperature before being subjected to substrate 3-amino-9-ethylcarbazole or 3,3′diaminobenzidine (Vector Laboratories, Burlingame, CA). The stained slides were photographed using a Nikon Eclipse 80i microscope equipped with a digital camera (DS-Ri1, Nikon, Shanghai, China).

### Indirect Immunofluorescence Staining Assay

For tissue indirect immunofluorescence staining, the staining method was the same as in (24). For HK2 cell indirect immunofluorescence staining, a total of 5 × 10^5^ cells were seeded into laser confocal dishes with a preplaced glass slide. Cells were treated by 50 ng/ml TNF-α with or without EVR for 24 h. Then, the cells were fixed with methanol for 15 min at room temperature and washed twice with PBS. After blocking was completed, sections were co-incubated with anti-FN (Abcam, USA), anti-E-cadherin (Abcam, USA), anti-p65 (CST, USA), anti-ubiquitin (Santa Cruz, USA) and anti-IκB-α (CST, USA) overnight at 4°C. Next, the cells were stained with 4,6-diamidino-2-phenylindole (DAPI) to visualize the nuclei and washed twice with PBS. Finally, cells were detected under a Nikon Eclipse 80i microscope (DS-Ri1, Nikon, Shanghai, China).

### Wound Healing and Transwell Migration Experiments

HK2 cells that had been treated with TNF-α were seeded into 6-well culture plates. For the wound-healing experiment, a straight scratch was made in the cell monolayer with a 200-μl pipette tip when the cells were 90% confluent. DMEM/F12 with 10% fetal bovine serum (FBS) was changed to serum-free DMEM/F12 (DFSF) for 24 h. The scratch was examined and photographed under a light microscope, and the cell-free area was quantified using the ImageJ software.

For the Transwell migration assay, the cells were seeded into the upper chamber of 24-well Transwell plates with 200 μl of medium. Then, the lower chamber was filled with 600 μl of 10% FBS-supplemented DFSF, and the HK2 cells were cultured for 12 h. Subsequently, the cells on the upper surface of the chamber were removed using a cotton swab. The cells on the bottom surface were fixed in 4% paraformaldehyde (PFA) for 30 min and stained with 0.1% crystal violet for 10 min. The migrated cells were photographed under a microscope, and the images were analyzed using ImageJ software.

### Cell Culture and Treatment

The HK2 cells were purchased from KeyGEN BioTECH (Nanjing, China). The cells were cultured in DFSF medium containing 10% FBS (Gibco, Carlsbad, CA, USA) at 37°C with 5% CO_2_, supplemented with 1% penicillin-streptomycin. HK2 cells were seeded on six-well culture plates for 24 h in complete medium containing 10% FBS. After starvation with DFSF for 16 h, the cells were modulated with TNF-α (50 ng/ml, Peprotech, Rocky Hill, NJ, USA) and/or EVR (5, 10, 50, and 100 nM, Selleck, Houston, TX, USA) for 48 h. In some experiments, cells were pretreated with CQ (20 μM, Selleck, Houston, TX, USA) or protein kinase B (Akt) inhibitor MK2206 (10 μM, Selleck, Houston, TX, USA) or QNZ (100 nM, Selleck, Houston, TX, USA) for 2 h before the addition of TNF-α and/or EVR. The cells were then collected for different assays. For small interfering RNA (siRNA) transfection assay, HK-2 cells at 80% confluence were transfected with Skp2-specific siRNA by using Lipofectamine 2000 (Invitrogen, Carlsbad, CA, USA) according to the instructions. Then, cells were treated as previously described after transfection for 6 h. The siRNAs were obtained from HanHeng Biotechnology (Shanghai, China). All the experiments were repeated at least three times.

### Western Blot and Coimmunoprecipitation Assay

Cells and kidney tissue were lysed by RIPA lysis buffer containing cocktail protease inhibitors for 30 min on ice. To isolate nuclear proteins, HK-2 cells were collected and nuclear were extracted according to the instruction of Nuclear and Cytoplasmic Extraction kit (KeyGEN BioTECH, Nanjing, China). Proteins from HK-2 cells and rat tissues were fractionated by electrophoresis on 8%–12% sodium dodecyl sulfate-polyacrylamide gel electrophoresis, electroblotted to polyvinylidene fluoride filter membranes, and incubated with the primary antibody at 4°C. For the co-IP experiment, briefly, the cell supernatants were pre-treated with protein A/G agarose (Santa Cruz Bio-technology, sc-2003) beads for 1 h at 4°C. Then, 2 μg of the corresponding primary antibody was added to the supernatants overnight at 4°C. Next, 50 μl of protein A/G agarose was added for 4 h. The beads were washed more than three times using ice-cold PBS or cell lysis buffer, and the bound proteins were boiled in loading buffer for further analysis. The primary antibodies were as follows: anti-β-actin (CST, Danvers, MA, USA), anti-Glyceraldehyde 3-phosphate dehydrogenase (GAPDH) (CST, Danvers, MA, USA), anti-Histone H3 (CST, Danvers, MA, USA), anti-β-tubulin (CST, Danvers, MA, USA), anti-E-cadherin (SAB Biotech, College Park, MD, USA), anti-α-SMA (SAB Biotech, College Park, MD, USA), anti-FN (BD Biosciences, USA), anti-Akt (CST, Danvers, MA, USA), anti-phospho-Akt (CST, Danvers, MA, USA), anti-mammalian target of rapamycin (mTOR) (CST, Danvers, MA, USA), anti-Unc-like kinase 1 (ULK1) (CST, Danvers, MA, USA), anti-phospho-mTOR (CST, Danvers, MA, USA), anti-phospho-P70S6K (CST, Danvers, MA, USA), anti-IκB Kinase α (IKKα) (CST, Danvers, MA, USA), anti-phospho-IKKα (CST, Danvers, MA, USA), anti-IκB-α (CST, Danvers, MA, USA), anti-phospho-IκB-α (CST, Danvers, MA, USA), anti-nuclear factor kappa B (NF-κB) p65 (CST, Danvers, MA, USA), anti-phospho-NF-κB p65 (CST, Danvers, MA, USA), anti-ubiquitin (Santa Cruz, USA), anti-Smurf1 (SAB Biotech, College Park, MD, USA), and anti-Smurf2 (SAB Biotech, College Park, MD, USA). This step was followed by incubation with an anti-rabbit or anti-mouse secondary antibody (1:4,000; ZSGB-BIO, Beijing, China). GAPDH, β-actin, and Histone H3 were used to verify equal loading of proteins. The intensity of the indicated bands were measured by Image Lab software (Bio-Rad, Hercules, CA, USA).

### Quantitative Real-Time Polymerase Chain Reaction

Total RNAs were extracted from tissues and cells with the RNA Extraction Kits (TIANGEN, Beijing, China). Detailed steps are described in (24). Gene expressions were measured by real-time PCR assay (Vazyme, Nanjing, China) and a DNA Engine Opticon 2 System (BioRad Laboratories, Hercules, CA, USA). Every experiment was repeated at least three times. The specific primers used were as follows:

NFKBIA: 5′‐CTCCGAGACTTTCGAGGAAATAC‐3′ (F)

5′‐GCCATTGTAGTTGGTAGCCTTCA‐3′ (R)

### Electron Microscopy

The samples were fixed with ice-cold glutaraldehyde (3% in 0.1 M cacodylate buffer, pH 7.4) and further processed by the Core Facility (Servicebio, Wuhan, China). Observations were performed on a JEOL JEM-2100 transmission electron microscope.

### Transcriptome Sequencing

Total RNA was used as input material for the RNA sample preparations. Sequencing libraries were generated using NEBNext^®^ UltraTM RNA Library Prep Kit for Illumina^®^ (NEB, USA) following manufacturer’s recommendations and index codes were added to attribute sequences to each sample.

Briefly, mRNA was purified from total RNA using poly-T oligo-attached magnetic beads. Fragmentation was carried out using divalent cations under elevated temperature in NEBNext First Strand Synthesis Reaction Buffer (5×). First strand cDNA was synthesized using random hexamer primer and M-MuLV Reverse Transcriptase (RNase H). Second-strand cDNA synthesis was subsequently performed using DNA Polymerase I and RNase H. Remaining overhangs were converted into blunt ends *via* exonuclease/polymerase activities. After adenylation of 3’ ends of DNA fragments, NEBNext Adaptor with hairpin loop structure was ligated to prepare for hybridization. In order to select cDNA fragments of preferentially 250–300 bp in length, the library fragments were purified with AMPure XP system (Beckman Coulter, Beverly, USA). Then 3 μl of USER Enzyme (NEB, USA) was used with size-selected, adaptor-ligated cDNA at 37°C for 15 min followed by 5 min at 95°C before PCR. Then, PCR was performed with Phusion High-Fidelity DNA polymerase, Universal PCR primers, and Index (X) Primer. Last, PCR products were purified (AMPure XP system) and library quality was assessed on the Agilent Bioanalyzer 2100 system.

### Statistical Analysis

GraphPad Prism 5.0 (GraphPad Software, Inc., La Jolla, CA, USA) was used for statistical analysis. Data were presented as means ± standard error of the mean (SEM) from at least three independent experiments. Comparison between and within multiple groups was performed using one-way analysis of variance followed by Student-Newman-Keuls test. *p*-values of <0.05 were considered significant.

## Data Availability Statement

The original contributions presented in the study are publicly available. This data can be found here: https://www.ncbi.nlm.nih.gov/PRJNA757419.

## Ethics Statement

The animal study was reviewed and approved by the Institutional Animal Care and Use Committee at Nanjing Medical University. Written informed consent was obtained from the owners for the participation of their animals in this study.

## Author Contributions

RT and MG supervised and conceived the project. ZG, CS, JT, and ZW designed and carried out most of the experiments. MZ and SF collected the samples of rats. LS, HC, ZH, and XJ analyzed the data. ZG and ZW made the figures. ZG and RT drafted and revised the paper. All authors contributed to the article and approved the submitted version.

## Funding

This work has been supported by the National Natural Science Foundation of China (grant numbers 82070769, 81870512, 81770751, 81570676, 81470981, and 81100532).

## Conflict of Interest

The authors declare that the research was conducted in the absence of any commercial or financial relationships that could be construed as a potential conflict of interest.

## Publisher’s Note

All claims expressed in this article are solely those of the authors and do not necessarily represent those of their affiliated organizations, or those of the publisher, the editors and the reviewers. Any product that may be evaluated in this article, or claim that may be made by its manufacturer, is not guaranteed or endorsed by the publisher.

## References

[1] Carney EF. Transplantation: Survival Benefit of Accepting a Diabetic Deceased Donor Kidney. Nat Rev Nephrol (2017) 13(8):444. doi: 10.1038/nrneph.2017.88 28603273

[2] El-Husseini A Aghil A Ramirez J Sawaya B Rajagopalan N Baz M. Outcome of Kidney Transplant in Primary, Repeat, and Kidney-After-Nonrenal Solid-Organ Transplantation: 15-Year Analysis of Recent UNOS Database. Clin Transplant (2017) 31(11):e13108. doi: 10.1111/ctr.13108 28881060

[3] Schinstock CA Stegall M Cosio F. New Insights Regarding Chronic Antibody-Mediated Rejection and Its Progression to Transplant Glomerulopathy. Curr Opin Nephrol Hypertens (2014) 23(6):611–8. doi: 10.1097/MNH.0000000000000070 25295960

[4] Goldberg RJ Weng FL Kandula P. Acute and Chronic Allograft Dysfunction in Kidney Transplant Recipients. Med Clin North Am (2016) 100(3):487–503. doi: 10.1016/j.mcna.2016.01.002 27095641

[5] Wang Z Han Z Tao J Wang J Liu X Zhou W. Role of Endothelial-to-Mesenchymal Transition Induced by TGF-Beta1 in Transplant Kidney Interstitial Fibrosis. J Cell Mol Med (2017) 21(10):2359–69. doi: 10.1111/jcmm.13157 PMC5618680 28374926

[6] Sutariya B Jhonsa D Saraf MN. TGF-Beta: The Connecting Link Between Nephropathy and Fibrosis. Immunopharmacol Immunotoxicol (2016) 38(1):39–49. doi: 10.3109/08923973.2015.1127382 26849902

[7] An C Jia L Wen J Wang Y. Targeting Bone Marrow-Derived Fibroblasts for Renal Fibrosis. Adv Exp Med Biol (2019) 1165:305–22. doi: 10.1007/978-981-13-8871-2_14 31399971

[8] Cruz-Solbes AS Youker K. Epithelial to Mesenchymal Transition (EMT) and Endothelial to Mesenchymal Transition (EndMT): Role and Implications in Kidney Fibrosis. Results Probl Cell Differ (2017) 60:345–72. doi: 10.1007/978-3-319-51436-9_13 28409352

[9] Di Gregorio J Robuffo I Spalletta S Giambuzzi G De Iuliis V Toniato E. The Epithelial-To-Mesenchymal Transition as a Possible Therapeutic Target in Fibrotic Disorders. Front Cell Dev Biol (2020) 8:607483. doi: 10.3389/fcell.2020.607483 33409282 PMC7779530

[10] Yang J Antin P Berx G Blanpain C Brabletz T Bronner M. Guidelines and Definitions for Research on Epithelial-Mesenchymal Transition. Nat Rev Mol Cell Biol (2020) 21(6):341–52. doi: 10.1038/s41580-020-0237-9 PMC7250738 32300252

[11] Blom JN Feng Q. Cardiac Repair by Epicardial EMT: Current Targets and a Potential Role for the Primary Cilium. Pharmacol Ther (2018) 186:114–29. doi: 10.1016/j.pharmthera.2018.01.002 29352858

[12] Andugulapati SB Gourishetti K Tirunavalli SK Shaikh TB Sistla R. Biochanin-A Ameliorates Pulmonary Fibrosis by Suppressing the TGF-Beta Mediated EMT, Myofibroblasts Differentiation and Collagen Deposition in In Vitro and *In Vivo* Systems. Phytomedicine (2020) 78:153298. doi: 10.1016/j.phymed.2020.153298 32781391 PMC7395646

[13] Wang Z Divanyan A Jourd’heuil FL Goldman RD Ridge KM Jourd’heuil D. Vimentin Expression Is Required for the Development of EMT-Related Renal Fibrosis Following Unilateral Ureteral Obstruction in Mice. Am J Physiol Renal Physiol (2018) 315(4):F769–80. doi: 10.1152/ajprenal.00340.2017 PMC6335003 29631355

[14] Zhao C Xu Z Wang Z Suo C Tao J Han Z. Role of Tumor Necrosis Factor-Alpha in Epithelial-to-Mesenchymal Transition in Transplanted Kidney Cells in Recipients With Chronic Allograft Dysfunction. Gene (2018) 642:483–90. doi: 10.1016/j.gene.2017.11.059 29174387

[15] Romanowska-Prochnicka K Felis-Giemza A Olesinska M Wojdasiewicz P Paradowska-Gorycka A Szukiewicz D. The Role of TNF-Alpha and Anti-TNF-Alpha Agents During Preconception, Pregnancy, and Breastfeeding. Int J Mol Sci (2021) 22(6):2922. doi: 10.3390/ijms22062922 33805757 PMC7998738

[16] Al-Lamki RS Mayadas TN. TNF Receptors: Signaling Pathways and Contribution to Renal Dysfunction. Kidney Int (2015) 87(2):281–96. doi: 10.1038/ki.2014.285 25140911

[17] Fasolo A Sessa C. Targeting mTOR Pathways in Human Malignancies. Curr Pharm Des (2012) 18(19):2766–77. doi: 10.2174/138161212800626210 22475451

[18] Fischer L Saliba F Kaiser GM De Carlis L Metselaar HJ De Simone P. Three-Year Outcomes in *De Novo* Liver Transplant Patients Receiving Everolimus With Reduced Tacrolimus: Follow-Up Results From a Randomized, Multicenter Study. Transplantation (2015) 99(7):1455–62. doi: 10.1097/TP.0000000000000555 26151607

[19] Mjornstedt L Schwartz Sorensen S von Zur Muhlen B Jespersen B Hansen JM Bistrup C. Renal Function Three Years After Early Conversion From a Calcineurin Inhibitor to Everolimus: Results From a Randomized Trial in Kidney Transplantation. Transpl Int (2015) 28(1):42–51. doi: 10.1111/tri.12437 25176389

[20] Shigematsu T Tajima S Fu R Zhang M Itoyama Y Tsuchimoto A. The mTOR Inhibitor Everolimus Attenuates Tacrolimus-Induced Renal Interstitial Fibrosis in Rats. Life Sci (2021) 288:120150. doi: 10.1016/j.lfs.2021.120150 34793770

[21] Eren MF Ay Eren A Sayan M Yucel B Elagoz S Ozguven Y. The Impact of Everolimus and Radiation Therapy on Pulmonary Fibrosis. Med (Kaunas) (2020) 56(7):348. doi: 10.3390/medicina56070348 PMC7404687 32668776

[22] Masola V Carraro A Zaza G Bellin G Montin U Violi P. Epithelial to Mesenchymal Transition in the Liver Field: The Double Face of Everolimus In Vitro. BMC Gastroenterol (2015) 15:118. doi: 10.1186/s12876-015-0347-6 26369804 PMC4570634

[23] Sarbassov DD Ali SM Sabatini DM. Growing Roles for the mTOR Pathway. Curr Opin Cell Biol (2005) 17(6):596–603. doi: 10.1016/j.ceb.2005.09.009 16226444

[24] Gui Z Suo C Wang Z Zheng M Fei S Chen H. Impaired ATG16L-Dependent Autophagy Promotes Renal Interstitial Fibrosis in Chronic Renal Graft Dysfunction Through Inducing EndMT by NF-kappaB Signal Pathway. Front Immunol (2021) 12:650424. doi: 10.3389/fimmu.2021.650424 33927720 PMC8076642

[25] Hill C Li J Liu D Conforti F Brereton CJ Yao L. Autophagy Inhibition-Mediated Epithelial-Mesenchymal Transition Augments Local Myofibroblast Differentiation in Pulmonary Fibrosis. Cell Death Dis (2019) 10(8):591. doi: 10.1038/s41419-019-1820-x 31391462 PMC6685977

[26] Pang M Wang H Rao P Zhao Y Xie J Cao Q. Autophagy Links Beta-Catenin and Smad Signaling to Promote Epithelial-Mesenchymal Transition *via* Upregulation of Integrin Linked Kinase. Int J Biochem Cell Biol (2016) 76:123–34. doi: 10.1016/j.biocel.2016.05.010 27177845

[27] Sun Y Xiong L Wang X Wang L Chen B Huang J. Autophagy Inhibition Attenuates TGF-Beta2-Induced Epithelial-Mesenchymal Transition in Lens Epithelial Cells. Life Sci (2021) 265:118741. doi: 10.1016/j.lfs.2020.118741 33181173

[28] Zhou J Cheng H Wang Z Chen H Suo C Zhang H. Bortezomib Attenuates Renal Interstitial Fibrosis in Kidney Transplantation *via* Regulating the EMT Induced by TNF-Alpha-Smurf1-Akt-mTOR-P70S6K Pathway. J Cell Mol Med (2019) 23(8):5390–402. doi: 10.1111/jcmm.14420 PMC6653435 31140729

[29] Liu Y Zhang Z Yan L Li X Zhang J Zhang X. Everolimus Reduces Postoperative Arthrofibrosis in Rabbits by Inducing Autophagy-Mediated Fibroblast Apoptosis by PI3K/Akt/mTOR Signaling Pathway. Biochem Biophys Res Commun (2020) 533(1):1–8. doi: 10.1016/j.bbrc.2020.08.039 32919704

[30] Choi YY Seok JI Hwang JI Kim DS. Co-Administration of Everolimus and N-Acetylcysteine Attenuates Hepatic Stellate Cell Activation and Hepatic Fibrosis. Am J Transl Res (2020) 12(6):2627–39. PMC7344068 32655795

[31] Tomei P Masola V Granata S Bellin G Carratu P Ficial M. Everolimus-Induced Epithelial to Mesenchymal Transition (EMT) in Bronchial/Pulmonary Cells: When the Dosage Does Matter in Transplantation. J Nephrol (2016) 29(6):881–91. doi: 10.1007/s40620-016-0295-4 27026415

[32] Ramadan R Faour D Awad H Khateeb E Cohen R Yahia A. Early Treatment With Everolimus Exerts Nephroprotective Effect in Rats With Adriamycin-Induced Nephrotic Syndrome. Nephrol Dial Transplant (2012) 27(6):2231–41. doi: 10.1093/ndt/gfr581 22036940

[33] Kurdi A De Doncker M Leloup A Neels H Timmermans JP Lemmens K. Continuous Administration of the Mtorc1 Inhibitor Everolimus Induces Tolerance and Decreases Autophagy in Mice. Br J Pharmacol (2016) 173(23):3359–71. doi: 10.1111/bph.13626 PMC5738667 27638766

[34] Nakagawa S Nishihara K Inui K Masuda S. Involvement of Autophagy in the Pharmacological Effects of the mTOR Inhibitor Everolimus in Acute Kidney Injury. Eur J Pharmacol (2012) 696(1-3):143–54. doi: 10.1016/j.ejphar.2012.09.010 23022334

[35] Wang Y Liu Z Shu S Cai J Tang C Dong Z. AMPK/mTOR Signaling in Autophagy Regulation During Cisplatin-Induced Acute Kidney Injury. Front Physiol (2020) 11:619730. doi: 10.3389/fphys.2020.619730 33391038 PMC7773913

[36] Zhang Q Lenardo MJ Baltimore D. 30 Years of NF-Kappab: A Blossoming of Relevance to Human Pathobiology. Cell (2017) 168(1-2):37–57. doi: 10.1016/j.cell.2016.12.012 28086098 PMC5268070

[37] Colleran A Ryan A O’Gorman A Mureau C Liptrot C Dockery P. Autophagosomal IkappaB Alpha Degradation Plays a Role in the Long Term Control of Tumor Necrosis Factor-Alpha-Induced Nuclear factor-kappaB (NF-Kappab) Activity. J Biol Chem (2011) 286(26):22886–93. doi: 10.1074/jbc.M110.199950 PMC3123056 21454695

[38] Shapira M Kakiashvili E Rosenberg T Hershko DD. The mTOR Inhibitor Rapamycin Down-Regulates the Expression of the Ubiquitin Ligase Subunit Skp2 in Breast Cancer Cells. Breast Cancer Res (2006) 8(4):R46. doi: 10.1186/bcr1533 16859513 PMC1779462

[39] Simone V Ciavarella S Brunetti O Savonarola A Cives M Tucci M. Everolimus Restrains the Paracrine Pro-Osteoclast Activity of Breast Cancer Cells. BMC Cancer (2015) 15:692. doi: 10.1186/s12885-015-1717-8 26468083 PMC4606500

[40] Li J Basler M Alvarez G Brunner T Kirk CJ Groettrup M. Immunoproteasome Inhibition Prevents Chronic Antibody-Mediated Allograft Rejection in Renal Transplantation. Kidney Int (2018) 93(3):670–80. doi: 10.1016/j.kint.2017.09.023 29229189

[41] Zhao H Watts HR Chong M Huang H Tralau-Stewart C Maxwell PH. Xenon Treatment Protects Against Cold Ischemia Associated Delayed Graft Function and Prolongs Graft Survival in Rats. Am J Transplant (2013) 13(8):2006–18. doi: 10.1111/ajt.12293 PMC3884761 23710625

