# Supplementary figures and images for: Everolimus Alleviates Renal Allograft Interstitial Fibrosis by Inhibiting Epithelial-to-Mesenchymal Transition Not Only via Inducing Autophagy but Also via Stabilizing IκB-α
Source: Front Immunol. 2022 Jan 24;12:753412. doi: 10.3389/fimmu.2021.753412 (PMC8818677; doi:10.3389/fimmu.2021.753412)

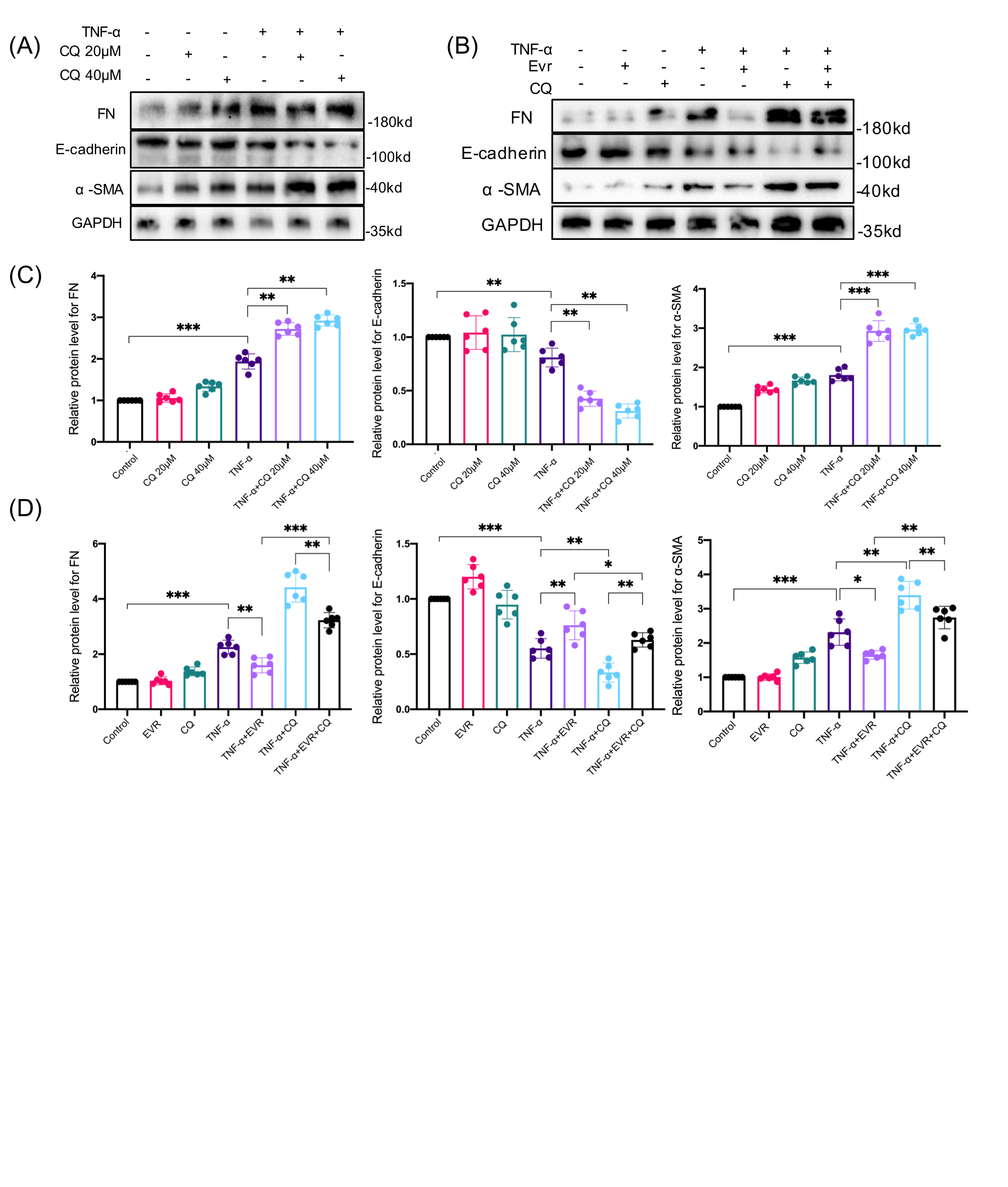

Supplement: Supplementary file 1 [file Image_1.tif]

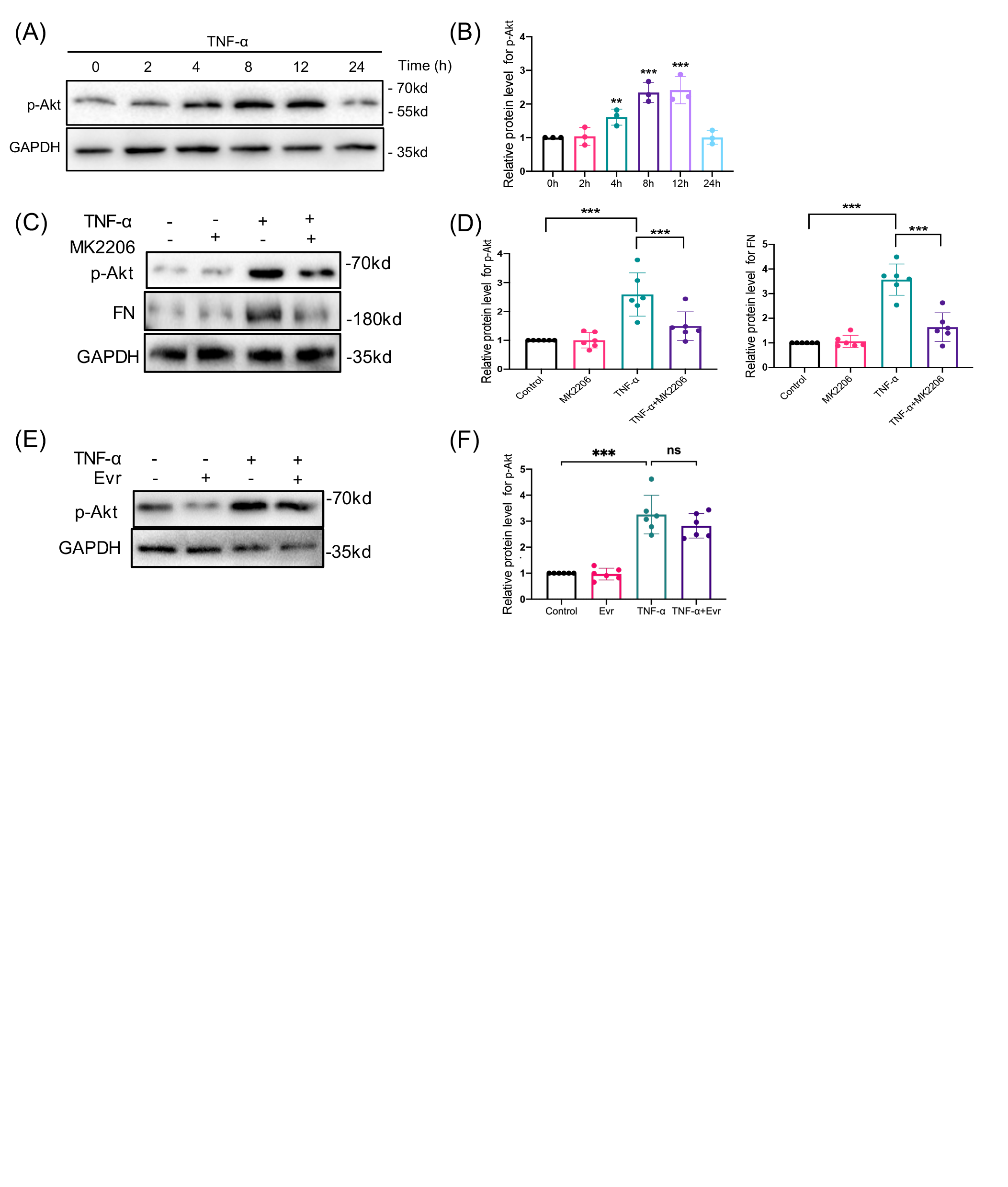

Supplement: Supplementary file 2 [file Image_2.tif]
